# Fabrication of 3D Bioprinted Bi-Phasic Scaffold for Bone–Cartilage Interface Regeneration

**DOI:** 10.3390/biomimetics8010087

**Published:** 2023-02-21

**Authors:** Hongyi Chen, Giovanni Gonnella, Jie Huang, Lucy Di-Silvio

**Affiliations:** 1 Department of Mechanical Engineering, University College London, London WC1E 7JE, UK; 2 Faculty of Dentistry, Oral & Craniofacial Sciences King’s College London, London SE1 1UL, UK

**Keywords:** bioprinting, cartilage regeneration, bone regeneration, 3D cell culture system

## Abstract

Treatments for osteochondral defects (OCDs) are mainly palliative and, with the increase in this pathology seen among both young and elderly people, an alternative treatment modality is sought. Many tissue-engineered strategies have been explored for regenerating the cartilage–bone interface; however, they generally fall short of being ideal. Although cell-laden hydrogel scaffolds are a common approach for bone and cartilage tissue regeneration, they usually lack homogenous cell dispersion and patient specificity. In this study, a biphasic 3D bioprinted composite scaffold was fabricated for cartilage–bone interface regeneration. To overcome the shortcoming of both materials, alginate–gelatin (A–G) hydrogel was used to confer a naturally occurring environment for the cells and polycaprolactone (PCL) was used to enhance mechanical stability, thus maximizing the overall performance. Hydroxyapatite fillers were added to the PCL in the bone phase of the scaffold to improve its bioactivity. Physical and biological evaluation of scaffolds in both phases was assessed. The scaffolds demonstrated a desirable biological response both singly and in the combined PCL/A-G scaffolds, in both the short term and longer term, showing promise as an interfacial material between cartilage and bone.

## 1. Introduction

Osteochondral tissues found in synovial joint surfaces consist of two parts—the articular cartilage and the subchondral bone. Articular cartilage principally acts to absorb shock and loads for the underlying subchondral bone, provides a lubricated surface for smooth joint motions, and contributes to joint stability [1,2,3]. The subchondral bone is a layer of lamellar bone lying immediately deep in the calcified zone of the articular cartilage, providing mechanical support for the cartilage by attenuating most impulsive loads on the joints [1,4,5]. The osteochondral interface tissue acts as a connecting structure for two diverse structures, each with a different composition and mechanical properties. Hence, it possesses complex physiological properties specific to each tissue, with the osteochondral tissue interfacing and subserving both. Within this space, both chondrocytes and osteoblasts respond to stimuli in a spatiotemporal manner, activating a biological response. Osteochondral tissue is approximately 3 mm thick in adults and has a hierarchical layered structure comprising cartilage, calcified cartilage, and subchondral bone [6].

Underneath the subchondral bone lies the subchondral trabecular bone, which is more porous and metabolically active compared with the subchondral bone plate. Articular cartilage consists of four zones: the superficial zone, the middle zone, the deep zone, and the calcified zone. The collagen fibers are aligned parallel to the articular surface in the superficial zone, obliquely in the middle zone, and perpendicular to the surface in the deep zone. The compression modulus of cartilage increases from the superficial zone to the calcified zone. In the calcified zone, collagen fibers are anchored into the subchondral bone perpendicularly, providing the interface bonding and mechanical transition between the bone and cartilage. This cartilage–bone interface maintains the integrity and function of the joint [7,8]. It is the complex nature of the cartilage–bone interface and the different healing capacity between one vascularized and one avascular tissue that makes it challenging to successfully regenerate the osteochondral lesion [9]. The different physiochemical properties of cartilage and subchondral bone are shown in Table 1.

Articular cartilage and meniscus can become damaged as a result of trauma, disease, or wear and tear of the joint. Furthermore, cartilage damage can cause further degeneration owing to the limited self-healing ability of cartilage resulting from its lack of blood supply [1,11,15,16,17]. As collagen fibers are not continuous between the calcified cartilage and the subchondral bone, the structure of the bone–cartilage interface is intrinsically weaker than the transitions within the cartilage zones [4]. Cartilage defects can expose the subchondral bone to higher stress and lead to subchondral bone defects. Such defects include both cartilage and bone and are common occurrences in osteoarthritis [18,19].

Tissue engineering has emerged as an alternative strategy for regenerating bone, cartilage, and the osteochondral tissue interface. Early osteochondral tissue engineering approaches including osteochondral autograft transfer and autologous chondrocyte implantation have been shown to regenerate cartilage and alleviate pain in early-term to midterm follow-up studies [20,21,22]. However, these approaches that focus on only cartilage have limitations of scar tissue formation, undesirable cartilage fills, limited integration between cartilage and subchondral bone, and long-term performance. To address these limitations, the osteochondral scaffold approach has been developed with the potential to regenerate the subchondral bone simultaneously with the regeneration of the overlying cartilage [23,24]. The osteochondral scaffolds mimic the 3D structure of both natural cartilage and bone tissue to facilitate cell attachment, proliferation, and regeneration of tissues. A major target for tissue engineering is to develop novel scaffolds with the capability of addressing specific requirements for different tissues such as cartilage and bone tissues [25].

For cartilage regeneration, hydrogels are rapidly gaining interest as scaffold material as they resemble the natural extracellular matrix and have been shown to facilitate chondrogenesis and regeneration of cartilage [26]. Furthermore, cells embedded in hydrogels have been shown to retain their normal physiological activity, owing to the 3D structure aiding cell adhesion, growth, proliferation, and maintenance of their natural phenotype. Among the different hydrogels, alginate has been extensively used owing to its high biocompatibility [27,28]. However, it lacks cell interaction properties. Gelatin, on the other hand, is not only a biodegradable and biocompatible polymer, but it also promotes cell attachment and signaling [29]. Hence, when combined with alginate, both material properties and biological interactions are enhanced [30].

For bone regeneration, porous bone scaffolds play an essential role by facilitating nutrient transportation; the attachment, migration, and proliferation of cells; and vascularization [31,32]. Polymer-based scaffolds have more recently been used for bone tissue engineering thanks to their biocompatibility, ductility, and tailorability in terms of their structural properties. Polycaprolactone, an FDA-approved polymer, has been widely used for bone tissue engineering. The semi-crystalline polycaprolactone presents a rubbery state at body temperature, resulting in its high toughness, elasticity, and mechanical strength compared with other polymers [33,34]. Bioactive fillers have been used to enhance the bioactivity and bone regeneration of PCL. Hydroxyapatite (HA) is a calcium phosphate mineral with a high chemical similarity to the inorganic component of human bone, and hence possesses a strong affinity to host bone tissues [35]. It has been widely used in bone tissue engineering and dental applications due to its bioactivity, biocompatibility, osteoconductivity, and osteoinductivity [36,37].

Three-dimensional (3D) printing has gained significant importance as a method for fabricating hydrogel scaffolds, with the potential to produce a tailored architecture in a cost-efficient manner [38,39]. A layer-by-layer processing method is preferred where the hydrogel is deposited one layer on top of another. For a viable 3D printed scaffold, cells can be encapsulated prior to printing for better spatiotemporal control [40]. For bone scaffolds, different techniques have been studied, one of which is direct ink writing (DIW). DIW is able to operate at room temperature, allowing a broad selection of biomaterials, temperature-sensitive drugs, and biomolecules to be used [41].

Many studies described in the literature have focused on cartilage scaffolds for osteochondral tissue engineering without addressing the need for the regeneration of subchondral bone. This can cause issues with integration and long-term performance. Engineering scaffolds to mimic both cartilage and bone with different properties for their regeneration simultaneously remains a great challenge in osteochondral tissue engineering. In this work, a hybrid osteochondral scaffold was fabricated by integrating several biofabrication techniques for the regeneration of the cartilage–bone interface. It consists of alginate–gelatin/PCL scaffold and hydroxyapatite-loaded PCL scaffold for cartilage and subchondral tissue regeneration, respectively. For the cartilage scaffold, chondrocytes were embedded in alginate–gelatin hydrogel composite bioink and bioprinted alongside a PCL framework 3D printed by the fused-deposition modelling (FDM) technique. The bioprinted structure yielded desirable swelling and degradation properties and high viability of chondrocytes. The hydroxyapatite-loaded PCL scaffold was 3D printed with the DIW technique. It has supported the adhesion and high viability of human osteoblast (HOB) cells. Furthermore, it facilitated the proliferation and mineralization abilities of HOB cells for at least 21 days.

## 2. Materials and Methods

### 2.1. Raw Materials

Sodium alginate (W201502), gelatin (type A, from porcine skin, ~300 g Bloom), calcium chloride (Mw = 110.98), Dulbecco’s modified Eagle medium (high glucose), and PCL pallets (molecular weight: 80 kDa) were purchased from Sigma-Aldrich. Low-viscosity sodium alginate was purchased from Alfa Aesar. Collagenase (type II, MW = 68–130 k) was purchased from Stemcell Technologies. Oxoid phosphate buffer saline tablets were purchased from Thermofischer. PCL filaments for FDM were purchased from eSun 3D. Hydroxyapatite microparticles were synthesized in the lab using the method previously reported [3]. Dichloromethane (DCM: CH_2_Cl_2_) was purchased from VWR Chemicals.

### 2.2. Bioink Preparation

Alginate and gelatin were chosen as materials for the bioinks because of their intrinsic complementary properties. In total, four different bioinks were created based on previous studies [42], including 5% alginate (A), 5% low-viscosity alginate (LVA), 5% alginate–3% gelatin (A–G), and 5% low-viscosity alginate–3% gelatin (LVA-G).

To generate the bioink stock solution, alginate was initially dissolved in sterile high glucose DMEM with the help of a magnetic stirrer heated to a temperature of 55 °C. Gelatin was subsequently added to the heated solution in order to achieve better homogenization. Stock solutions were finally transferred to 50 mL falcon tubes and centrifuged for 10 min at a rotation speed of 3000 rpm to remove the presence of bubbles formed during the stirring process. Any foam presence was subsequently removed with the aid of a spatula. Gel solutions were finally stored at 2 °C until further use. As a crosslinker, 100 mM CaCl_2_ solution was prepared by dissolving calcium chloride powder in deionized water and then sterilizing via autoclave. This solution was then stored at 2 °C until further use. Before printing, the stock solutions were heated to 30 °C. Once liquid, 3 mL of solution was placed in a 3 mL syringe and used to resuspend the previously centrifuged cells. To remove any air bubbles, the syringe was finally centrifuged at 300 rpm for 2 min, without affecting the cell dispersion.

To make the PCL polymer-based ink, 50% w/v of PCL was dissolved in DCM under magnetic stirring. Then, 30 wt% of hydroxyapatite microparticles was added to the PCL pure ink to enhance its viscosity and bioactivity. The inks were centrifuged for 5 min at a speed of 3000 rpm to remove bubbles before 3D printing.

### 2.3. Degradation Rate

The degradation rate of the printed scaffolds was evaluated in both hydrolytic (PBS) and enzymatic (32 mg collagenase type II/200 mL PBS) conditions. Scaffolds were dried and weighed to measure the initial weight ($W_{0}$). The samples were then submerged in either PBS or collagenase for 1, 3, 7, 14, and 21 days. At each time point, the gels were dried, frozen overnight at −20°C, lyophilised, and weighed ($W_{t}$). The degradation was calculated as follows:

(1) $$Degradation\;\left(\%\right)=\frac{W_{t}-W_{0}}{W_{0}}*100\%$$

### 2.4. Rheological Assessments

Rheology tests were performed using the Discovery-2 rheometer. Flow sweep tests were conducted on bioinks and PCL ink with the shear rate ranging from 0.1 s^−1^ to 100 s^−1^. The temperature of 20 °C was used for rheological tests on PCL inks, while 24 °C and 31 °C were used for rheological tests on bioinks.

### 2.5. Scaffold Fabrication

The hydrogel scaffolds were generated using the Regemat3D extrusion-based bioprinter, using 3 cc heated syringes and 0.41 mm needles. The printing speed was set to 6 mm/s, while the printing temperature was set at 31 °C. A pore size of 0.2 mm was chosen prior to printing, together with a layer thickness of 0.35 mm. A diameter of 12 mm and a height of 2.1 mm were used based not only on multi-well dimensions (24-well plates can accommodate a 12 mm disc), but more importantly to reflect the average cartilage thickness, ranging from 1.7 to 2.55 mm [43]. Scaffolds were initially printed in 98 mm glass Petri dishes, submerged in 100 mM CaCl_2_ for 20 min on each side, and finally washed in PBS for 10 min.

Porous 0°/90° PCL cylindric scaffolds were printed with the fused deposition modelling (FDM) technique using a thermoplastic extruder (E3D V6, 0.4 mm nozzle, 1.75 mm filament) on Regemat3D. Samples were printed following the same dimensions as for the hydrogels (12 mm in diameter and 3.2 mm in height), with a fibre thickness of 0.35 mm and fibre distance of 0.9 mm.

**Figure 1:**
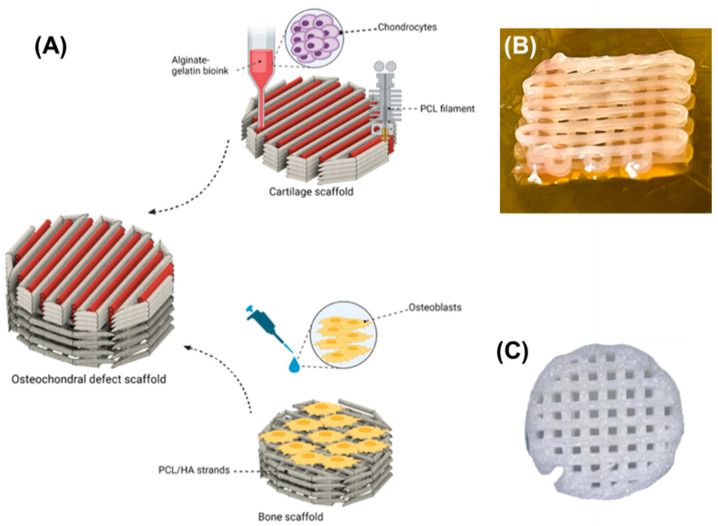
(**A**) Schematics demonstrating the incorporation of the co-printed alginate–gelatin hydrogel and PCL scaffold and DIW printed PCL/HA composite scaffold into a hybrid osteochondral scaffold for bone–cartilage interface regeneration. (**B**) Co-printed alginate/gelatin hydrogel and PCL scaffold for cartilage regeneration. (**C**) Three-dimensional (3D) printed PCL/30HA composite scaffold with the DIW technique for subchondral bone regeneration.

The PCL/A-G co-printed scaffolds were produced by printing PCL filaments and filling the gaps with hydrogel bioink in a layer-by-layer manner, as shown in Figure 1A. In this way, optimal cell distribution and gel deposition were achieved. The same settings used to produce the PCL and the hydrogel scaffolds were used for these samples. PCL scaffold matrix was first printed with the FDM technique to build a mechanical framework and the chondrocyte-embedded hydrogel bioink was subsequently deposited into the gaps of the PCL filaments.

The PCL-based scaffolds for subchondral bone were 3D printed with a DIW printer modified from a commercial FDM printer, Prusa i3. The nozzle diameter was 0.6 mm, the printing speed was 10 mm/s, and the printing temperature was 20 °C. Scaffold structures and ribbons with 0° and 90° orientations were 3D printed for cell culture. The diameter and height of the DIW printed PCL-based scaffold are around 10 mm and 3 mm, respectively.

### 2.6. Biological Characterisation

#### 2.6.1. In Vitro Cell Culture

Normal human knee articular chondrocytes (Lonza bioscience, Cat. No: CC-2550) were cultured in high glucose Dulbecco’s modified Eagle medium containing 20% fetal bovine serum, 1% penicillin–streptomycin, 1% L-glutamine, and 0.05% ascorbic acid at 37 °C with 5% CO_2_. The medium was changed every 72 h. Passage 23 cells were used for this study, with a total of 6 × 10^6^ cells/mL during bioprinting.

Furthermore, 1 × 10^5^ chondrocytes were microseeded on top of PCL-based scaffolds printed using the FDM technique (previously sterilised in 70% EtOH).

Human osteoblast (HOB) cells (PromoCell Cat. No: C-12720) were cultured with HOB culture media. HOB media was Dulbecco’s modified Eagle’s medium (DMEM; Hyclone, UT, USA) supplemented with 10% fetal bovine serum (Hyclone) and 1% penicillin–streptomycin (Hyclone). Then, 1 × 10^5^ HOB cells were suspension-seeded on each scaffold.

#### 2.6.2. Scaffold Cytotoxicity

MTT (3-(4,5-Dimthylthiazol-2-yl)-2,5-Diphenyltetrazolium Bromide) was used to measure cellular metabolic activity [44]. Owing to their robustness and rapid turnover, human osteosarcoma (HOS) cells (Saos-2 cell line, Sigma Aldrich, Cat. No: 89050205) were used for this first screening of the bioinks. In brief, cell-free printed samples were initially placed in elution for both 24 and 72 h on a roller shaker in osteoblast media (four samples per hydrogel group were used). HOS cells were seeded at 10^4^ cells/well in two 96-well plates and incubated for 24 h. The cells were subsequently incubated with the eluted media for a further 24 and 72 h prior to the addition of MTT solution (25 mg of MTT was dissolved in 5 mL, which was diluted in ascorbic-free osteoblast medium). The plates were incubated for 4 h, after which they were gently blotted dry, and 100 mL of dimethyl sulfoxide (DMSO) was added to each well. Plates were positioned on a plate shaker for 5 min before placing them in a microplate reader. The absorbance of the samples was estimated at a wavelength of 570 nm. As controls, cells cultured in osteoblast media (positive control) and in osteoblast media with 10% EtOH (negative control) were used.

#### 2.6.3. Viability Assessment

The viability of chondrocytes and HOB cells was assessed using the Live/Dead Viability/Cytotoxicity Kit (L-3244) from Invitrogen. Staining solutions were made by mixing calcein AM (1 μL/mL) and ethidium homodimer-1 (1 μL/mL) in culture media. Live cells were labelled with calcein-AM, producing green fluorescence, while dead cells were labelled with ED-1, emitting red fluorescence [45]. Specimens were submerged in the staining solution and imaged using an epifluorescence microscope (Olympus IX51 Microscope). The number of live and dead cells was quantified using Fiji [46].

#### 2.6.4. Proliferation Assessment

Proliferation assessment was conducted using alamarBlue^TM^ assay, as previously described [47,48]. Here, 1 × 10^5^ chondrocytes were bioprinted per sample in a density of 2 × 10^6^ cells/mL in the A-G bioink and 1 × 10^5^ HOB cells were seeded per PCL-based scaffold. The proliferation of chondrocytes in A–G bioink was assessed up to 14 days after bioprinting and HOB cells seeded on DIW printed PCL-based scaffold were assessed up to 21 days. Cell-seeded scaffolds were placed in a 48-well plate with culture medium for the respective test time periods. At each time point, existing media was removed and replaced with the 10% alamarBlue^TM^ solution and scaffolds were incubated for 4 h in total. The negative control was 10% alamarBlue^TM^ solution with no cells and the tissue culture control was cells on tissue culture plastic. Following incubation, 100 μL of media from each of the 48 wells was taken and placed in a 96-well plate, and the absorbance was read using a microplate reader at a test wavelength of 570 nm and reference wavelength of 630 nm (Dynex Technologies, Chantilly, VA, USA). The results showed the absorbance rate between day 1 and day 14 in the alginate–gelatin bioprinted scaffolds.

#### 2.6.5. Cell Mineralization Assessment

Calcium deposition on the HOB seeded 3D printed scaffolds was determined by Alizarin red S staining of HOB cells cultured on the scaffolds at day 21. On the day of measurement, the scaffolds were washed with PBS, fixed with 10% formal saline, and then washed with deionised water. The fixed specimens were stained with 1 mL 2% Alizarin red S for 15 min and then washed with 50% ethanol. Images of the stained scaffolds were taken for qualitative assessment. Quantitative assessments were conducted by adapting a previously published protocol [49]. The bound stain on the scaffolds was dissolved with 1 mL of 10% cetylpyridium chloride (CPC) solution. Aliquots (100 μL) of the supernatant were read using a microplate reader (Opsys MR™ 96-well microplate reader, Dynex Technologies, Chantilly, VA, USA) at 570 nm. Serial dilutions of known concentrations of Alizarin red S in CPC solution were read under the microplate reader to obtain the standard curve of Alizarin red S. Cell mineralization results were expressed as μ moles of calcium per well as 1 mole of Alizarin red S binds to 2 moles of calcium in an Alizarin red S-calcium complex [50]. Measurements from the blank control (unseeded scaffolds) were subtracted from the calcium deposition measurements of seeded scaffolds.

#### 2.6.6. Cell Morphology

SEM was performed to assess cell morphology. Following an in-house established protocol, the samples were prepared for imaging, leaving them overnight in 0.1 M sodium cacodylate trihydrate containing 1.5% glutaraldehyde and performing multiple washes in different concentrations of EtOH. Samples were then air-dried and sputter coated with gold palladium before imaging them with the SEM microscope (JCM-7000 Neoscope Benchtop SEM, EM ACE600 sputter coater).

### 2.7. Statistical Analysis

Statistical analysis was performed with one-way analysis of variance (ANOVA) with Tukey’s post hoc multiple comparison test. A *p*-value smaller than 0.05 was considered as statistically significant.

## 3. Results and Discussions

### 3.1. Physical Characterizations of Hydrogel Bioinks

**Figure 2:**
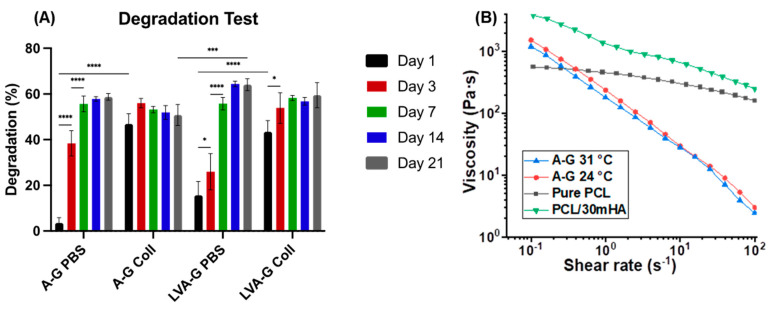
(**A**) Degradation test of the alginate–gelatin bioinks with PBS and collagenase for up to 21 days. * *p* < 0.05, *** *p* < 0.001, **** *p* < 0.0001. (**B**) Shear viscosity profile of the A−G bioink at 24 °C and 31 °C, and pure PCL ink and PCL/30HA ink for DIW at room temperature.

The addition of gelatin in both alginate and low viscosity alginate hydrogels prevented their disintegration during the swelling test. A–G and LVA–G had swelling ratios of 165% and 155% at room temperature at day 21, as shown in Figure S1. This proves their capability of supporting the transport of nutrients and waste products for embedded cells. Degradation tests showed that the scaffolds degraded in both PBS and collagenase, as expected (Figure 2A). Scaffolds degrade faster in collagenase and reached equilibrium faster (day 1 vs. day 7 for A–G and day 3 vs. day 14 for LVA–G). A–G bioink demonstrated significantly lower degradation at day 21 than LVA–G (50% vs. 64%) in PBS (*p* < 0.001) and has maintained its structural integrity (Figure S2). A-G was thus selected for further biological characterizations owing to its desirable physical properties.

### 3.2. Rheological Properties

Shear viscosity profiles of A–G hydrogel at two temperatures, pure PCL and PCL/30HA, are reported in Figure 2. All inks show shear-thinning behaviour, which is desired for 3D printing as it facilitates extrusion and can safeguard cells from excess shear stress during extrusion. For A–G ink, the increase in temperature from 24 °C to 31 °C decreases the viscosity. Here, 31 °C was used as the printing temperature as it is closer to the physiological temperature and the viscosity is lower, leading to a less restrictive environment for embedded chondrocytes. For PCL-based ink for DIW, the addition of HA significantly increased the viscosity. The fillers can restrain the long-range motion of polymer chains, increasing the shear resistance and thus the viscosity of the ink [51]. The addition of fillers also decreased the solvent content in the ink, and thus increased the viscosity.

### 3.3. Biological Properties

#### 3.3.1. Bioink Cytotoxicity Assessment

**Figure 3:**
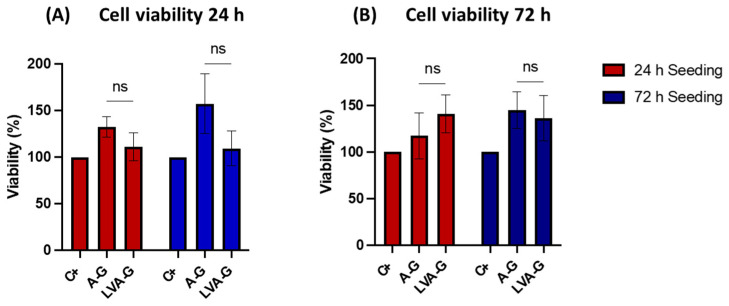
Cell viability of chondrocytes when in contact with the eluants from the scaffolds. The chondrocyte cells were cultured for a total of 24 h (**A**) and 72 h (**B**). No significant difference was observed between the LVA–G and A–G groups.

MTT assay was used to assess the viability of HOS cells exposed to the elutes of the bioinks for 24 h and 72 h. The MTT results showed optimal retention of cell viability in the A–G group, which reached a peak of 157% and 144% of cell viability when cultured for 24 h and 72 h in the 72 h eluted medium, respectively (Figure 3). The LVA–G gel showed good cell viability as well, with a peak of 135% when cells were cultured for 72 h in the 72 h eluted medium. Even though no significant differences between gel solutions were found, A–G seemed to release more cell-friendly nutrients. No significant differences were detected between the positive control and hydrogel solutions.

#### 3.3.2. Cell Viability Assessments

**Figure 4:**
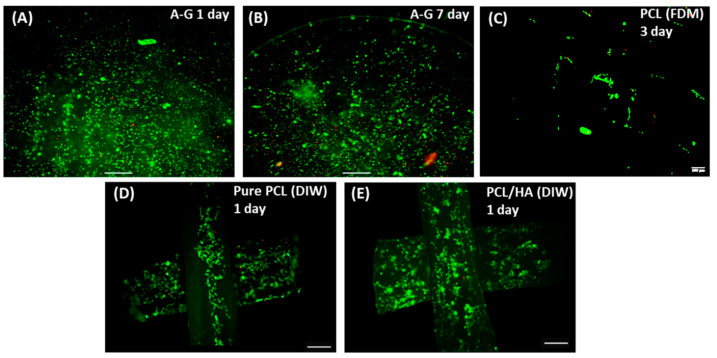
Confocal images showing viability live/dead assay for chondrocytes and HOB cells in the bi-phasic scaffold. Chondrocytes embedded in A–G bioink were assessed 1 day (**A**) and 7 days (**B**) post-bioprinting, and chondrocytes seeded on FDM printed PCL (**C**) were assessed after culturing for 3 days (scale bar: 500 μm). HOB cells seeded on the DIW printed pure PCL scaffold (**D**) and PCL/HA scaffold (**E**) were assessed after culturing for 1 day (scale bar: 200 μm).

The viability of chondrocytes 1 day and 7 days after bioprinting and after 3 days of culture on PCL scaffold was assessed with live/dead assay, as shown in Figure 4. The viability was 84% after 24 h, increasing up to 87% on day seven, indicating that the A–G bioink provided a desirable environment for the proliferation of chondrocytes (Figure 4A,B). Chondrocyte viability on the PCL printed scaffolds was estimated to be 81% after 3 days (Figure 4C). Live/dead staining was performed on the HOB cells cultured on 3D printed porous scaffolds after 24 h, as shown in Figure 4. Cells on both the top and second layers of the scaffolds were imaged. Cells were observed throughout the different layers of all scaffolds, confirming the interconnectivity of pores and migration of cells down to the lower layers of the scaffold. The high viability of HOB cells on both pure PCL scaffold (>96%) and PCL/30HA scaffold (>98%) confirmed the biocompatibility of the scaffolds.

#### 3.3.3. Cell Proliferation

**Figure 5:**
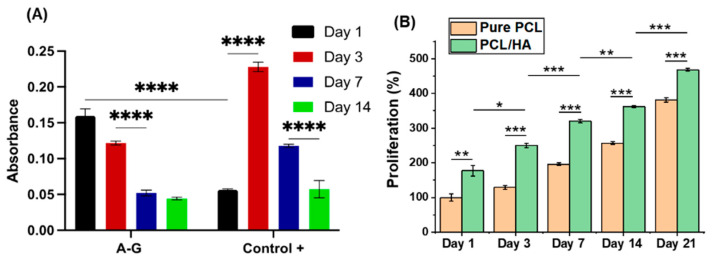
(**A**) The proliferation of chondrocytes in A–G bioink over 1–14 days. (**B**) Proliferation of HOB cells cultured for up to day 21 on pure PCL and PCL/30HA scaffolds. The results are expressed as a percentage of PCL on day 1. Statistically significant differences are shown as * *p* < 0.05, ** *p* < 0.01, *** *p* < 0.001, and **** *p* < 0.0001.

AlamarBlue^TM^ proliferation assay was performed to assess the proliferation of chondrocytes in A–G bioink after bioprinting and HOB cells seeded on DIW printed PCL-based scaffold—Figure 5. For chondrocytes in the bioprinted A–G bioink, surprisingly, no increase in cell proliferation was observed during this period (Figure 5A). Cell activity peaked on day one, followed by a decrease on day 3 and day 7. No significant changes were observed from day 7 to day 14. Chondrocytes in the control group also decreased from day 3, day 7, and day 14 when the absorbance level was not statistically different compared with the A–G group. The decrease in metabolic activity does not necessarily indicate a decrease in viability as the live/dead assay showed a high viability of chondrocytes in the A–G bioink at day 14 (Figure S3). A possible explanation is that chondrocytes were too densely distributed and had reached a stage of contact inhibition in both the A–G bioink and cell control group. The proliferation of HOB cells cultured on the scaffolds is shown in Figure 5B. The results were normalized to PCL on day 1. The proliferation of HOB cells on all scaffolds increased as a function of time until day 21 (up to 520%). This confirmed that the PCL-based scaffolds provided a desirable environment for the long-term growth and proliferation of HOB cells. The addition of HA in the PCL scaffold resulted in a significant increase in the proliferation of HOB cells at all time points, indicating an enhanced cell activity, also confirmed by the viability results.

#### 3.3.4. Mineralization Assessment

**Figure 6:**
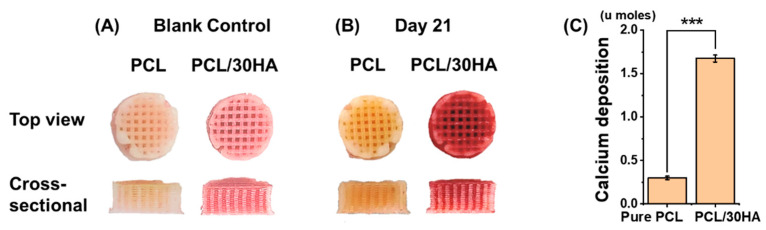
Mineralization assessment of HOB cells cultured in the 3D printed PCL-based scaffolds for up to day 21 using Alizarin red S assay. Qualitative assessment was shown with images of top and cross-sectional views of Alizarin red staining on the blank control (**A**) and HOB seeded scaffolds after 21 days of culturing (**B**). (**C**) Quantitative assessment of calcium deposition in HOB seeded PCL-based scaffolds. The results are reported as u moles per well/scaffold (*** *p* < 0.001).

Calcium deposition of suspension-seeded HOB cells on 3D printed PCL-based scaffolds was assessed by Alizarin red S assay with both qualitative and quantitative assessments, as shown in Figure 6. The qualitative results show enhanced red staining across the scaffold cultured with HOB cells for 21 days, confirming that cells deposited mineralized matrix throughout the scaffold (Figure 6A,B). The results confirmed the interconnectivity of the pores in the scaffold, which allow cells to migrate, attach, and proliferate, as well as the bioactivity of the scaffolds inducing mineralization. The interconnectivity of pores can also contribute to the long-term biocompatibility of the scaffolds as it provides a higher surface area and volume in the scaffold for cell activity and facilitates nutrients’ and waste diffusion. Quantitative assessments were also conducted on the DIW printed PCL-based scaffolds. The absorbance reading of scaffolds was compared to the standard curve (Figure S4) to measure the Alizarin red S concentration and then calculate the calcium deposition. The quantitative results further confirmed the bioactivity of incorporated hydroxyapatite in PCL scaffolds Figure 6C). A significant increase in mineralization was observed in PCL/30HA scaffolds compared with the pure PCL scaffolds (*p* < 0.001), which confirms the bioactivity enhancement with the addition of fillers. This is also consistent with the proliferation measurement.

#### 3.3.5. Cell Morphology

**Figure 7:**
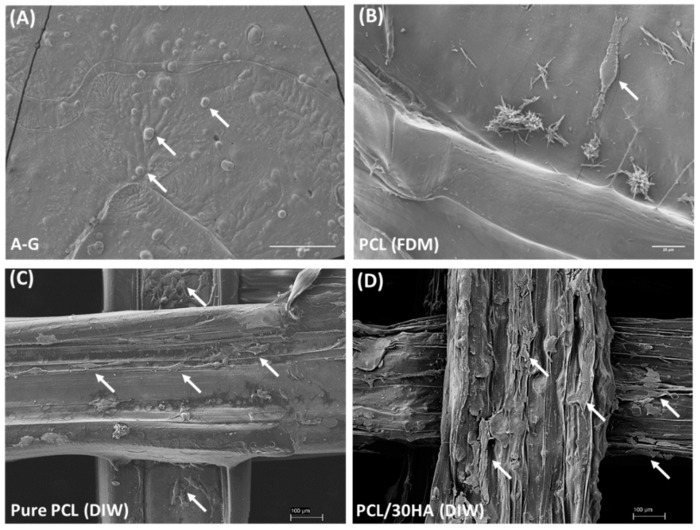
SEM images showing the morphology of chondrocytes and HOB cells in the bi-phasic scaffold. (**A**) Chondrocytes on the surface of bioprinted A–G bioink (scale bar 100 μm). (**B**) Chondrocytes seeded on PCL scaffold imaged at day 7 (scale bar 20 μm). HOB cells attached on the top two layers of DIW printed pure PCL (**C**) and PCL/30HA (**D**) porous scaffolds after 24 h of culture are shown (scale bar 100 μm). Cells are pointed out with arrows.

SEM images of chondrocytes and HOB cells in the bi-phasic scaffold are shown in Figure 7. It was observed that, following bioprinting, chondrocytes were able to retain their rounded morphology in the A–G bioink with a dimension in the range from 10 to 20 μm (Figure 7A). This resembles their shape in the natural extracellular matrix, indicating that the A–G bioink provided a favorable microenvironment for the chondrocytes. Chondrocytes were also observed to have adhered to the FDM printed PCL matrix of the co-printed cartilage scaffold. HOB cells were observed to successfully attach and spread on the different layers of the DIW printed PCL-based scaffolds (Figure 7C,D), confirming the biocompatibility and bioactivity of the scaffolds. The HOB cells exhibited improved spreading on the PCL/30HA scaffold, indicative of improved bioactivity with the addition of HA in PCL.

## 4. Conclusions

This study successfully integrated 3D bioprinting, FDM, and DIW techniques for the innovative fabrication of bi-phasic osteochondral scaffold for cartilage–bone interface regeneration. For the cartilage phase, the bioprinting of A–G hydrogel was coordinated with the 3D printing of PCL with FDM to co-print the A–G/PCL hybrid scaffold. The bioprinted A–G hydrogel supported the encapsulation of chondrocytes with high density, viability (84%), and proliferation for up to 21 days. The co-printed A–G/PCL hybrid scaffold facilitated the attachment of chondrocytes with high viability (>90%), proving its potential for cartilage regeneration. For the bone phase, the 3D printed PCL/30HA composite scaffold facilitated the diffusion, attachment, and spreading of HOB cells throughout the scaffolds with high viability (>96%), proliferation (up to 520%) for at least 21 days, and mineralization throughout all scaffolds. This demonstrates the importance of the interconnectivity of pores in the PCL/30HA scaffold and the potential for cells to migrate within and produce bone matrix for bone regeneration.

In summary, this study shows the integration of different biofabrication techniques combining the individual properties of each material to create a bi-phasic scaffold. The materials were selected as they are able to mimic the natural physiological niches for both chondrocytes and osteoblasts and support their biological and physical requirements, with the potential to regenerate both chondrogenic and osteogenic tissue. The encouraging results have laid the foundation for future work of fabricating a seamless continuous gradient scaffold, with the ability to regenerate the osteochondral interface defect in its entirety.

## Figures and Tables

**Table 1:** The biochemical composition and mechanical properties of cartilage and subchondral bone in natural osteochondral tissue.

Tissues	Cells	Main Chemical Compositions	Elastic/Young’s Modulus
Cartilage (superficial, middle, and deep zone) [1,7,8,10,11,12,13]	Chondrocytes	Dry weight: ~60 wt% Collagen (~90% Type II) ~35 wt% Proteoglycan (Water: 65–75 wt%)	0.1–2.0 MPa
Cartilage (Calcified zone) [13,14]	Chondrocytes (hypertrophic)	Dry weight: ~ 20 wt% Collagen (Type II) ~65 wt% Hydroxyapatite	6.44 ± 1.02 MPa
Subchondral bone [10,11,12,13,14,15,16]	Osteoblasts Osteoclasts Osteocytes Mesenchymal stem cells	Dry weight: ~ 80 wt% Hydroxyapatite ~ 10 wt% Collagen (>90% Type I) (Water: ~10 wt%)	297–475 MPa

## Data Availability

Raw data to reproduce the described findings are available upon request.

## Supplementary Materials

The following supporting information can be downloaded at: https://www.mdpi.com/article/10.3390/biomimetics8010087/s1. Figure S1. Dynamic swelling degree of the hydrogels without cells tested at room temperature (RT) and 37 °C. Figure S2. 3D printed A–G scaffold after 21 days of degradation test in collagenase (A) and PBS (B). Figure S3. Live staining of chondrocyte cells in A–G bioink after bioprinting for 14 days in the alamarBlue assay (scale bar: 500 μm). Figure S4. Standard curve of the absorbance of Alizarin red S solutions with a series of dilutions for measuring the calcium content deposited by HOB cells in the DIW printed scaffolds.
