# Supplementary material for: Fabrication of 3D Bioprinted Bi-Phasic Scaffold for Bone–Cartilage Interface Regeneration
Source: Biomimetics (Basel). 2023 Feb 21;8(1):87. doi: 10.3390/biomimetics8010087 (PMC10046269; doi:10.3390/biomimetics8010087)
Supplement: Supplementary file 1 [file biomimetics-08-00087-s001.zip › biomimetics-2142294-supplementary.pdf]

biomimetics

MDPI

Supplementary materials

Fabrication of 3D bioprinted bi-phasic scaffold for bone-cartilage interface regeneration

Hongyi Chen[1†], Giovanni Gonnella[2†], Jie Huang[1,*], and Lucy Di Silvio[2,*].

[1]Department of Mechanical Engineering, University College London, London, UK
[2]Faculty of Dentistry, Oral & Craniofacial Sciences King's College London, London, UK
*Correspondence, joint: Lucy Di Silvio Tel.: +44 207848 8475, Jie Huang Tel:+44 207679 7183
†These authors contributed equally to this work.

1. Physical characterisation methods for the hydrogel scaffolds

1.1. Swelling degree

Five (n=5) printed scaffolds were tested under two swelling conditions, at room temperature (T=25°C) for the equilibrium degree of swelling (EDS) and 37°C for the dynamic degree of swelling (DDS). Briefly, samples were initially dried overnight in a 37°C humidified chamber to record their initial dry weight (W_0). Consequently, they were kept in a solution of PBS for 1, 3, 6, and 24 hours. At each time point, samples were removed from the solution, dried on tissue paper, and the swelling weight (W_t) was recorded. The following equation (REF9) was used to assess both swelling degrees, in percentage:

$$Swelling\ (\%) = \frac{W_t - W_0}{W_0} * 100\%$$

2. Physical properties of hydrogel scaffolds

2.1. Dynamic swelling degree

All four groups of hydrogels showed high retention liquid at both temperatures tested. As seen in Figure S1, the gelatin-free groups reached the highest swelling degree, independently from the temperature (peak reached by the A group at 37°C of 274%). Although they swelled significantly less (the A-G group reached a maximum of 165% when at 37°C), the gelatin-loaded samples showed higher stability throughout the experiment, with alginate only scaffold showing significant deterioration in the 24-hours timeframe. The Anova test showed a significant difference between the A and LVA groups compared to A-G and LVA-G groups. On the other hand, no difference was observed between A-G and LVA-G groups at RT or 37°C. Hence, their swelling ability was not affected by temperature changes, which is key for in-vivo applications. For the previously mentioned reasons, gelatin-free samples were discarded from further tests.

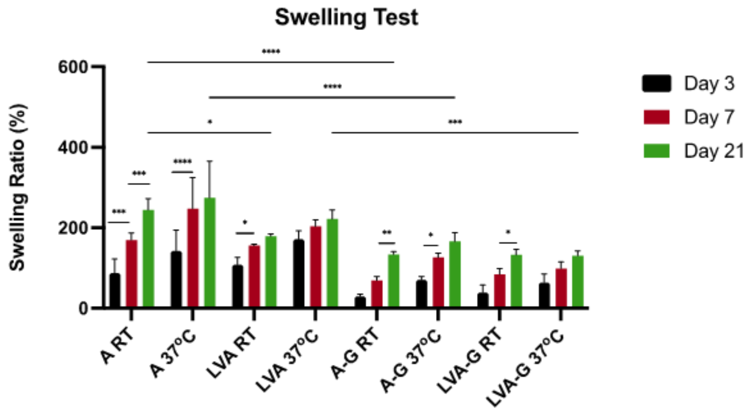

Figure S1 Dynamic swelling degree of the hydrogels without cells tested at room temperature (RT) and 37°C. Significant differences were found between A and LVA samples compared to gelatin-loaded samples. *$P<0.05$, **$p<0.01$, ***$p<0.001$, ****$p<0.0001$.

2.2. Scaffold stability

To further investigate gelatin-loaded scaffold stability, a 21-day degradation test was carried out. Figure S2AB shows the A-G scaffold after 21 days of degradation, with a noticeable shrinkage due to weight loss, but still structurally intact. Thus, based on this finding the A-G gel was used for bioprinting.

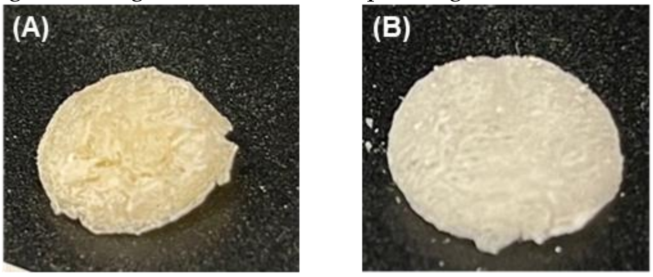

Figure S2 3D printed A-G scaffold after 21 days of degradation test in collagenase (A) and PBS (B).

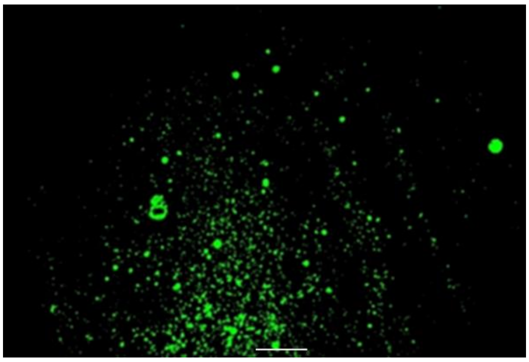

Figure S3 Live staining of chondrocyte cells in A-G bioink after bioprinting for 14 days in the alamarBlue assay (scale bar: 500 µm).

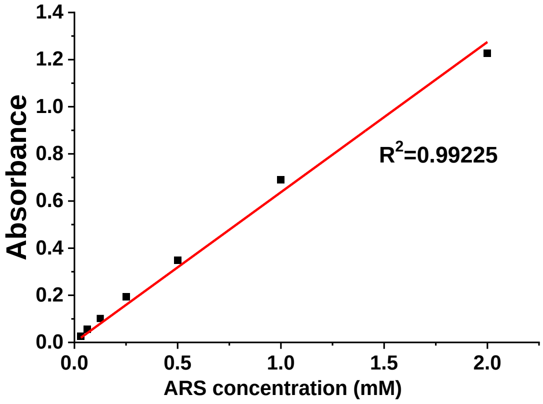

Figure S4 Standard curve of the absorbance of Alizarin red S solutions with a series of dilutions for measuring the calcium content deposited by HOB cells in the DIW printed scaffolds.
